# APPLIED GERONTOLOGY: HOW STUDENTS ADDRESS LONELINESS IN THE LOCAL COMMUNITY

**DOI:** 10.1093/geroni/igz038.901

**Published:** 2019-11-08

**Authors:** Eric Schoenmakers

**Affiliations:** Fontys University of Applied Sciences, Eindhoven, Netherlands

## Abstract

Fontys University of Applied Sciences offers an Undergraduate program in Applied Gerontology. Full-time and part-time students are trained in gerontology and in applying this knowledge in developing and implementing products and services in order to improve quality of life of older adults. In our vision, students learn in practice. Therefore, the educational program largely consists of authentic projects for real organizations in the local community. In one of these ongoing projects, students research loneliness in the local community. A network of organizations, which communicates intensively with the University, is involved. For these organizations, students study how clients and employee’s think about (coping with) loneliness, write testimonials about interventions, and advise in improving the services of organizations. For students, these kind of projects offer opportunities to learn in practice. For the community, students’ involvement means extra manpower to further develop services and society as a whole.

